# Click-correlative light and electron microscopy (click-AT-CLEM) for imaging and tracking azido-functionalized sphingolipids in bacteria

**DOI:** 10.1038/s41598-021-83813-w

**Published:** 2021-02-22

**Authors:** Simon Peters, Lena Kaiser, Julian Fink, Fabian Schumacher, Veronika Perschin, Jan Schlegel, Markus Sauer, Christian Stigloher, Burkhard Kleuser, Jürgen Seibel, Alexandra Schubert-Unkmeir

**Affiliations:** 1 Institute for Hygiene and Microbiology, Julius-Maximilian University Wuerzburg, Wuerzburg, Germany; 2 Institute for Organic Chemistry, Julius-Maximilian University Wuerzburg, Wuerzburg, Germany; 3 Institute of Pharmacy, Freie Universität Berlin, Berlin, Germany; 4 Imaging Core Facility, Biocenter, Julius-Maximilian University Wuerzburg, Wuerzburg, Germany; 5 Department of Biotechnology and Biophysics, Biocenter, Julius-Maximilian University Wuerzburg, Wuerzburg, Germany; 6 Department of Toxicology, University of Potsdam, Nuthetal, Germany; 7 Institute of Molecular Biology, University of Duisburg-Essen, Essen, Germany

**Keywords:** Imaging, Microbiology techniques, Microscopy, Biological techniques, Microbiology, Antimicrobials

## Abstract

Sphingolipids, including ceramides, are a diverse group of structurally related lipids composed of a sphingoid base backbone coupled to a fatty acid side chain and modified terminal hydroxyl group. Recently, it has been shown that sphingolipids show antimicrobial activity against a broad range of pathogenic microorganisms. The antimicrobial mechanism, however, remains so far elusive. Here, we introduce ‘click-AT-CLEM’, a labeling technique for correlated light and electron microscopy (CLEM) based on the super-resolution array tomography (srAT) approach and bio-orthogonal click chemistry for imaging of azido-tagged sphingolipids to directly visualize their interaction with the model Gram-negative bacterium *Neisseria meningitidis* at subcellular level. We observed ultrastructural damage of bacteria and disruption of the bacterial outer membrane induced by two azido-modified sphingolipids by scanning electron microscopy and transmission electron microscopy. Click-AT-CLEM imaging and mass spectrometry clearly revealed efficient incorporation of azido-tagged sphingolipids into the outer membrane of Gram-negative bacteria as underlying cause of their antimicrobial activity.

## Introduction

Sphingolipids, including ceramides, form a diverse group of structurally related lipids and are composed of a backbone of sphingoid bases coupled to a fatty acid side chain. A broad range of different head motives and complex glycosylation pattern results in further variability^1^. Besides their important function as bio-effector molecules, which are involved in the regulation of various aspects of cell growth, proliferation, and in anti-cancer therapeutics^2^, several studies indicate a role of sphingoid bases and fatty acids in the defense against pathogenic microorganisms^3–5^. The antimicrobial properties of host-derived lipids have become increasingly recognized in the past^6–8^ and their use for anti-infective therapy and prophylactic approaches seems attractive. Human lipids with antimicrobial properties include free fatty acids, monoglycerides and sphingolipids, showing different degrees of antimicrobial activity against Gram-positive and Gram-negative bacteria^3,4,8–13^. Whereas a recent study identified the mechanism of the bactericidal effect of sphingosine, by interaction with the bacterial membrane lipid cardiolipin leading to membrane permeabilization^14^, the antimicrobial mechanism of azido-modified sphingolipids is still unknown. A recent study demonstrated the antimicrobial activity of a synthetic ceramide analog against the Gram-negative bacterium *Neisseria meningitidis,* a major cause of septicemia and bacterial meningitis worldwide^15^. Here the addition of a functional azido group to the omega position of the fatty acid chain side of a synthetic short chain ceramide-resulting in ω-azido-C_6_-ceramide [ω-N_3_-C_6_-ceramide, Cer (d18:1/6:0-ω-N_3_)]-induced efficient killing of *N. meningitidis*^13,16^. Importantly, the compound showed no cytotoxic effects on eukaryotic cells^13^. However, it remains elusive if the observed antimicrobial effects are based on the interaction of the modified sphingolipid with the bacterial membrane, or accumulation in the cytoplasm where it might interfere with the microbial metabolism.

Various imaging methods can be used to analyze the mode of action of antimicrobial agents and complement biophysical studies. The combination of high-resolution imaging with fluorescence imaging and refined labeling techniques paves the way for tracking and quantification of an antimicrobial compound throughout the cell.

In this study, we established a protocol based on a combination of correlative light and electron microscopy (CLEM), i.e. super-resolution array tomography (srAT)^17,18^ and click chemistry^19^, which we call in short ‘click-AT-CLEM’. This combination of fluorescence and electron imaging applied here to the Gram-negative bacterium *N. meningitidis* allowed to determine morphological and ultrastructural changes after treatment with azido-modified sphingolipid analogs as well as to visualize their subcellular localization. A dibenzocyclooctyne (DBCO)-containing fluorescent dye (Alexa Fluor 488 DIBO analog (AFDye 488 DBCO AF)) was used for fluorescent labeling of azido-tagged sphingolipid analogs^13,16,20–22^ via Cu(I)-free strain-promoted alkyne-azide cycloaddition click chemistry reaction (SPAAC)^23,24^. Our data demonstrate that srAT technology in combination with click chemistry–based labeling reaction is ideally suited to visualize, track and quantify azido-modified antimicrobial sphingolipids in bacteria.

## Results

### Inhibitory and bactericidal activity of a novel sphingolipid analog, ω-N_3_-sphingosine, against *N. meningitidis*

We have recently documented minimal inhibitory and bactericidal concentration (MIC and MBC) values for the antimicrobial sphingolipid sphingosine as well as for short-chain and long-chain ceramides and several ceramide analogs, including α-N_3_-C_6_-ceramide, ω-N_3_-C_6_-ceramide, α-N_3_-C_16_-ceramide and ω-N_3_-C_16_-ceramide, against the Gram-negative microorganism *N. meningitidis* by broth microdilution assays and time killing studies^13^. The data demonstrated a potent bactericidal activity of sphingosine and the synthetic ceramide analog ω-N_3_-C_6_-ceramide against *N. meningitidis*^13^*.* Here, we now determined the MIC and MBC values for a newly synthesized sphingosine analog, ω-N_3_-sphingosine^22^, against *N. meningitidis*. ω-N_3_-sphingosine displayed a MIC value of 4 µg/ml and a MBC value of 8 µg/ml comparable to MIC/MBC values observed for the unmodified sphingosine for *N. meningitidis* (Table S1)^13^. In line with our previous study, we included *Escherichia coli* and *Staphylococcus aureus* as control organisms and MIC/MBC values of 16 µg/ml (MIC)/16 µg/ml (MBC) (for *E. coli*) and 8 µg/ml (MIC)/16 µg/ml (MBC) (for *S. aureus*) were determined (Table S1).

### Functionalized sphingolipids induce concentration dependent membrane alteration and disruption in *N. meningitidis*

**Figure 1:**
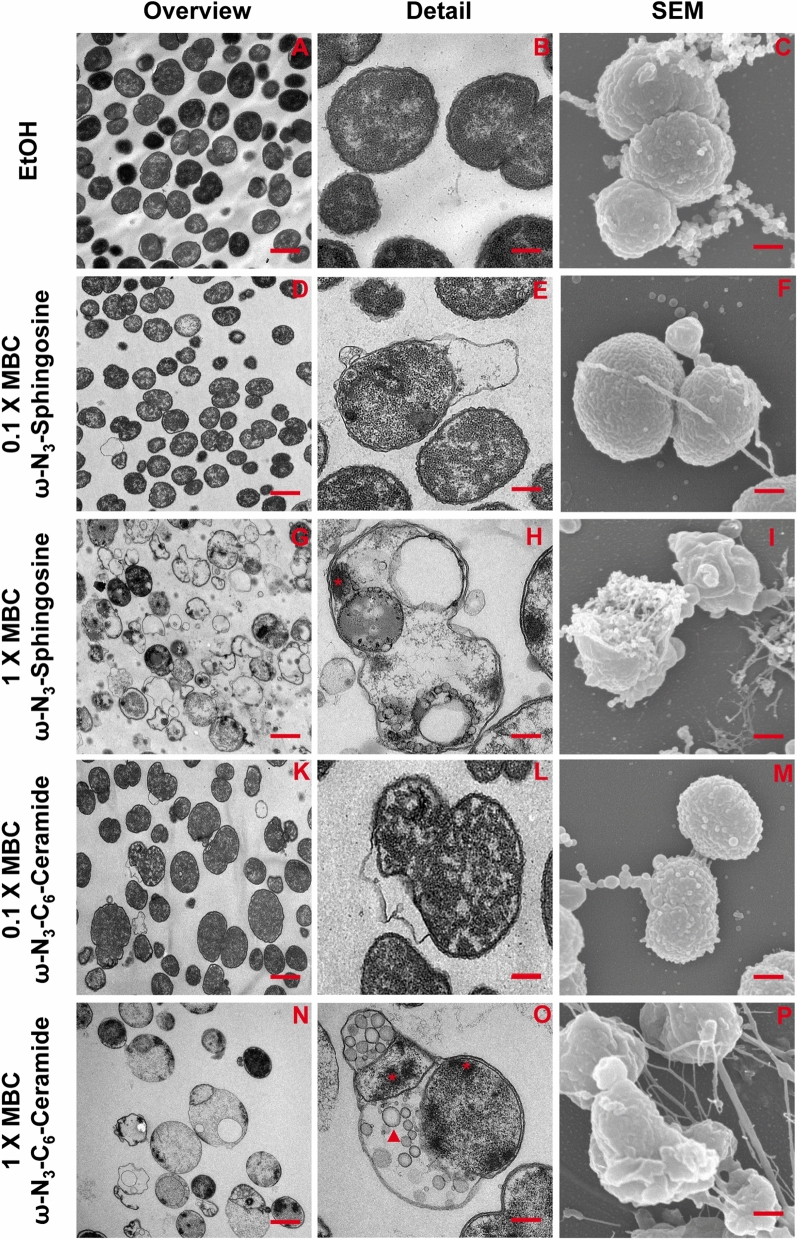
TEM and SEM micrographs show the effect of *N. meningitidis* treated with azido-modified sphingolipids. Representative TEM or SEM images of *N. meningitidis* treated with either EtOH (**A**–**C**), 0.1/1 × MBC of ω-N_3_-sphingosine (**D**–**F**/**G**–**I**) or 0.1/1 × MBC of ω-N_3_-C_6_-ceramide (**K**–**M**/**N**–**P**). *N. meningitidis* treated with 0.1 × MBC of ω-N_3_-sphingosine or ω-N_3_-C6-ceramide showed a slight alteration of their surfaces and shape (**F**,**M**) and intact intracellular content. However, some bacteria displayed a strong elongation of the outer membrane (**E**,**L**). *N. meningitidis* treated with 1 × MBC of ω-N_3_-sphingosine or ω-N_3_-C_6_-ceramide were distorted to various degrees and their surfaces were wrinkled (**I**,**P**). Many bacteria appeared in various stages of lysis with compromise of the cell wall and plasma membrane (**G**–**I**,**N**–**P**). Bacteria showed intracellular inclusion bodies appearing in high electron density (**H**,**O**, indicated with asterisks) in electron micrographs and additional vesicles that filled the intracellular content (**O**, indicated with arrowheads). First column: TEM overview images, scale bars 1 µm. Second column: TEM detail images, scale bars 0.25 µm. Third column: SEM detail images, scale bars 0.25 µm.

To investigate the effects of two azido-modified sphingolipids, ω-N_3_-sphingosine and ω-N_3_-C_6_-ceramide, on *N. meningitidis*, bacteria were treated with different amounts of the two compounds and analyzed by scanning electron microscopy (SEM) and transmission electron microscopy (TEM). Azido-modified sphingolipids allowed imaging of sphingolipids in prokaryotes^13^ via conventional fluorescence microscopy^20^ or high-resolution microscopy^13^. As control, *N. meningitidis* were treated with ethanol (solvent control). Ethanol treated control *N. meningitidis* exhibited coccus morphology with typical ‘kidney or coffee-bean shape’ and SEM showed the characteristic pairs of *N. meningitidis* cells forming a diplococcus (Fig. 1C). Control bacteria showed a slight increase in their electron density by TEM, but no morphological changes compared to non-solvent treated bacteria (Fig. 1A–C, Fig. S1). In contrast, *N. meningitidis* treated with a functionalized ω-N_3_-sphingosine or a ω-N_3_-C_6_-ceramide were distorted to various degrees and their surfaces were wrinkled when treated with a concentration corresponding to 1 × MBC (Fig. 1G–I, N–P). Bacteria showed an increased number of external blebs in SEM sections. In addition, bacteria appeared in various stages of lysis with compromised cell wall and plasma membrane. Bacteria treated with a lower concentration of the functionalized sphingolipids corresponding to 0.1 × MBC showed only a slight alteration of their surfaces and shape compared to control *N. meningitidis*.

In thin sections, control *N. meningitidis* showed the typical coccus morphology, the outer and inner membrane and the release of outer membrane vesicles were visible (Fig. 1A-B). In *N. meningitidis* treated with 0.1 × MBC of either ω-N_3_-sphingosine or ω-N_3_-C_6_-ceramide, some cells were still intact with an outer and inner membrane (Fig. 1D–F, K–M). In some bacteria, there was intact intracellular content, however with strong elongation of the outer membrane (Fig. 1E). *N. meningitidis* treated with 1 × MBC of both compounds showed different stages of disintegration and lysis (Fig. 1G,H,N,O). The cytoplasm was not uniform with flocculation and aggregation of intracellular contents. Bacteria showed intracellular inclusion bodies appearing in high electron density in electron micrographs and additional vesicles that filled the intracellular content (Fig. 1O).

We also investigated the effects of both azido-modified sphingolipids on *E. coli* und *S. aureus* by TEM analysis. Control *S. aureus* had a typical ‘coccus’ morphology and formed grape-like clusters (Fig. S2). *S. aureus* treated with ω-N_3_-C_6_-ceramide were unaffected and showed intact cell walls and the cytoplasmatic membranes were clearly visible (Fig. S2G,H). In line with this finding, when *E. coli* were treated with ω-N_3_-C_6_-ceramide bacteria were also unaffected, showed intact cell wall and membranes and the cytoplasm had a uniform granularity (Fig. S2E,F). In contrast, both *S. aureus* and *E. coli* treated with 1 × MBC of ω-N_3_-sphingosine were distorted to various degrees. ω-N_3_-sphingosine-treated *E. coli* showed electron dense intracellular inclusion bodies and additional vesicles that filled the intracellular content (Fig. S2A,B). Like that seen in *E. coli*, *S. aureus* treated with ω-N_3_-sphingosine also contained electron dense intracellular content and, in some bacteria, the remaining cell wall and cytoplasm were not intact and an aggregation of flocculation of intracellular content appeared (Fig. S2C,D).

### Click-AT-CLEM imaging reveals localization of azido-functionalized sphingolipids in *N. meningitidis*

While SEM/TEM-analysis allowed us to visualize the morphological and ultrastructural changes of the membrane and cell wall, we aimed to use a labeling protocol to track and quantify the sphingolipid distribution at subcellular level. As this is very challenging with established immunolabeling approaches for electron microscopy, we developed a novel pre-embedding correlative light and electron microscopy (CLEM) protocol to achieve a more precise localization of the uptake of the two azido-modified sphingolipids, ω-N_3_-sphingosine and ω-N_3_-C_6_-ceramide, by *N. meningitidis.* Recently, we successfully demonstrated that the toolkit of azido-modified sphingolipids can be used for imaging sphingolipids in mammalian cells^20^ and prokaryotes^13^ via conventional fluorescence microscopy^20^ or high-resolution microscopy^13^. Super-resolution array tomography (srAT)^17,18,25,26^ is an antibody-based staining CLEM workflow that combines preparation of the samples for super-resolution microscopy (e.g. structural illumination microscopy (SIM)) followed by electron microscopy, LR-White embedding and preparation of 100 nm ultrathin sections. Staining with sphingolipid specific antibodies, to detect only the incorporated sphingolipids, then takes place on the array of serial sections. Due to the embedding step prior to labeling, only a proportion of the epitopes are accessible to the staining protocol with antibodies, whereas a larger number of epitopes can be hidden in the resin making them inaccessible for the antibody^27^.

**Figure 2:**
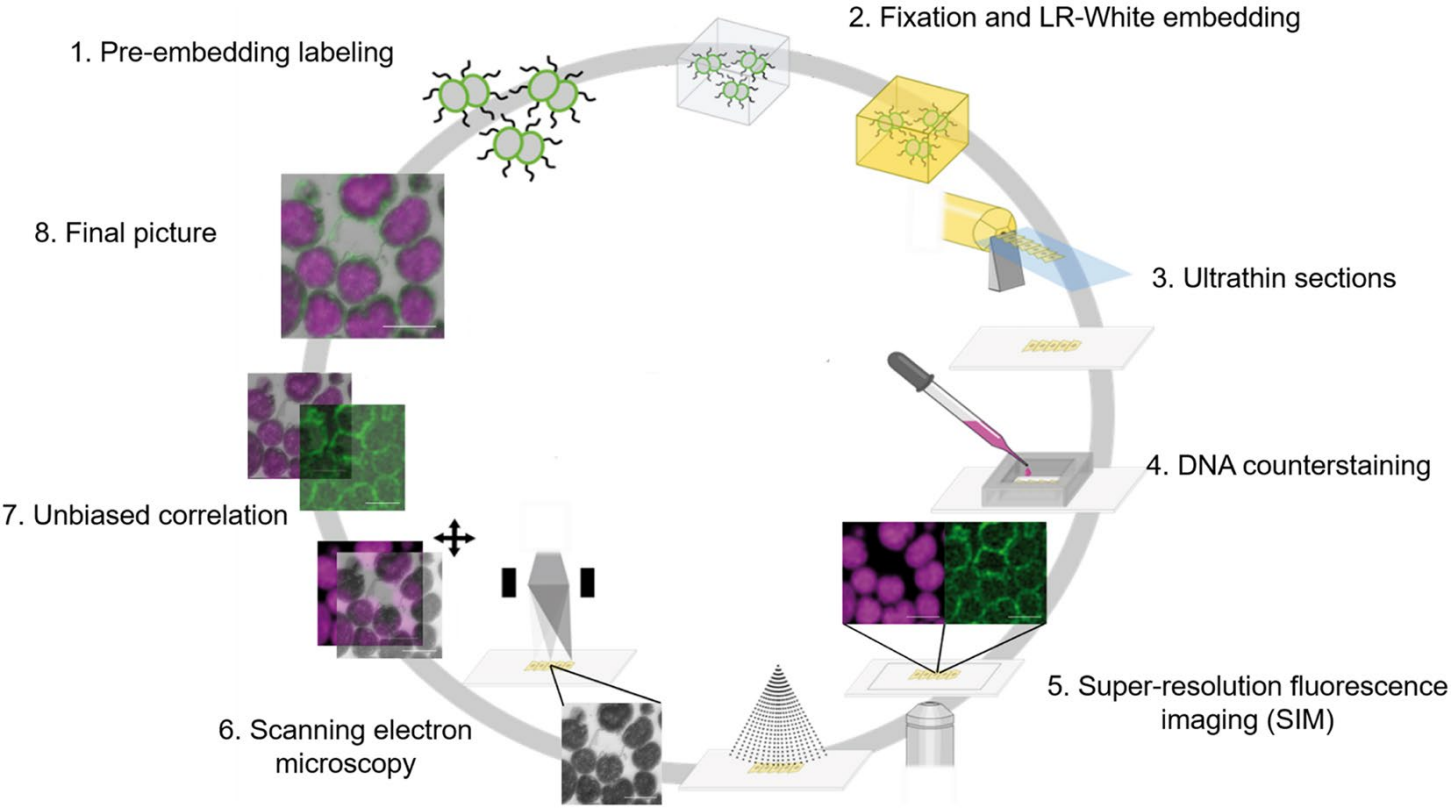
Scheme of the click-AT-CLEM workflow. The scheme shows a breakdown of most important steps in the click-AT-CLEM workflow used in this study. Green: lipids; magenta: DNA; grey: SEM. Scheme was adapted from Markert et al.^17^.

In contrast, azido-functionalized sphingolipid analogs can be visualized and tracked directly by fluorescence microscopy. To overcome this limitation, we used the functional azido group coupled to the ω-position of sphingosine or the amine-bound fatty acid side chain of ceramides, respectively, utilizing a copper-free click chemistry reaction. A dibenzocyclooctyne (DBCO)-containing fluorescent dye [Alexa Fluor 488 DIBO analog (AFDye 488 DBCO AF)] was used for the fluorescent labeling, that has been tested to preserve its fluorescence embedded in the LR-White resin, thus allowing CLEM for super-resolution fluorescence analysis followed by EM preparation protocols. Due to the pre-embedding labeling, the entire cell surface was accessible for direct labeling with the dye and not only the parts of the cell membrane facing the section surface as it would be the case in post-embedding on-section staining. In addition, click chemistry allowed us to specifically stain only the sphingolipids, which becomes especially important due to the lack of lipid specific antibodies (Fig. 2).

**Figure 3:**
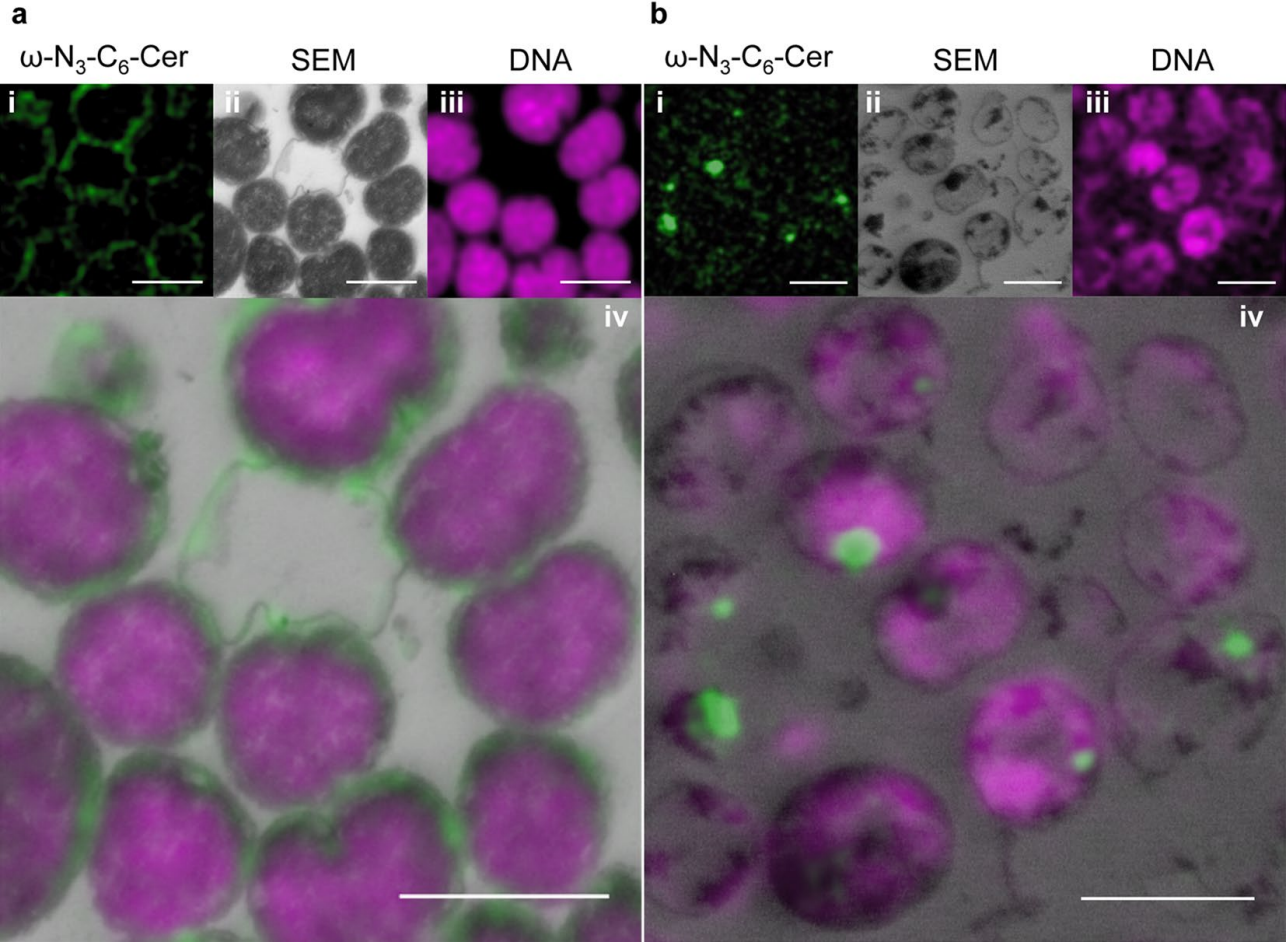
Click-AT-CLEM strategy reveals localization of ω-N_3_-C_6_-Ceramid in *N. meningitidis*. (**a**,**b**) Representative CLEM images of *N. meningitidis* treated with 0.1 × MBC (**a**) or 1 × MBC (**b**) of ω-N_3_-C_6_-ceramide. Upper rows show representative single images of the ω-N_3_-C_6_-ceramide [ω-N_3_-C_6_-Cer (green)] and DNA signal (magenta) taken with SIM (**i**,**iii**) and *N. meningitidis* image taken with SEM (**ii**). Final images after unbiased correlation are shown in (**iv**). Scale bars 1 µm.

To test the applicability of copper-free click chemistry for CLEM, bacteria were treated with different concentrations of ω-N_3_-C_6_-ceramide or ω-N_3_-sphingosine and labeled with AFDye 488 DBCO AF. Control cells that were not treated with functionalized sphingolipid analogs exhibited only minimal levels of unspecific background fluorescence after labeling with the dye (Fig. S3). Treatment of *N. meningitidis* with 0.1 × the MBC of ω-N_3_-C_6_-ceramide and subsequent labeling with AFDye 488 DBCO AF showed a similar morphological pattern by CLEM and only a subset of bacteria was affected (Fig. 3a-ii). Whereas the majority of the bacteria still displayed an unaltered ‘kidney or coffee-bean shape’, some bacteria showed elongations of their outer membrane (Fig. 3a-ii). In addition, all bacteria showed an unaffected cytosolic compartment with even dense content, which could also be seen on DNA staining images (Fig. 3a-iii). Interestingly, for some bacteria CLEM imaging revealed that dye-labeled ω-N_3_-C_6_-ceramide clearly integrated into the bacterial membrane and was found especially in the elongated outer membrane (Fig. 3a-i,iv). In contrast, treatment of *N. meningitidis* with 1 × the MBC of ω-N_3_-C_6_-ceramide and subsequent dye labeling resulted in distortion of the bacteria to various degrees and condensation of electron dense material in the cytosol of bacteria (Fig. 3b-ii). Moreover, dye-labeled ω-N_3_-C_6_-ceramide could not be detected in the bacterial membrane (Fig. 3b). CLEM imaging revealed that the electron dense material clearly correlated with accumulation of the labeled sphingolipid analog.

**Figure 4:**
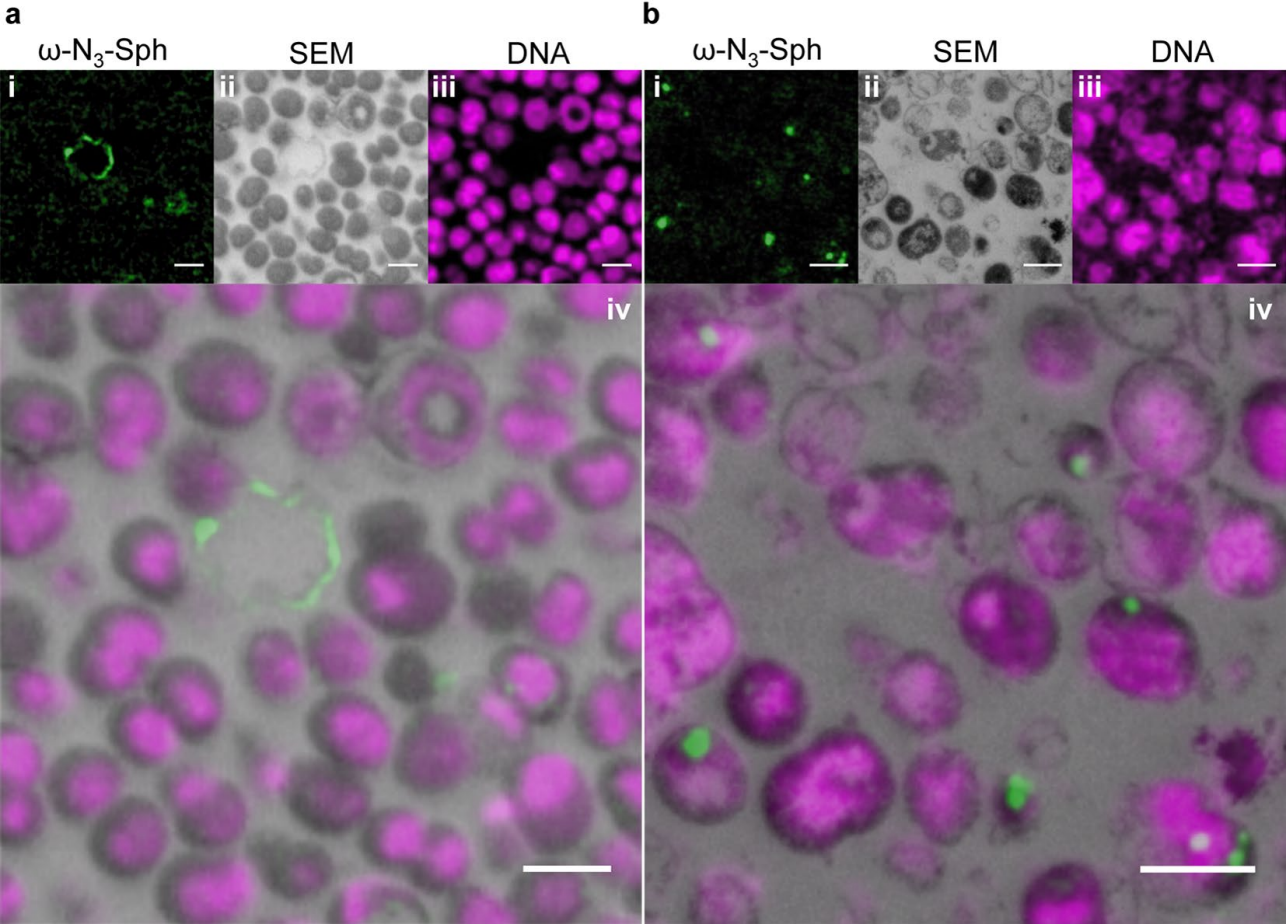
Click-AT-CLEM strategy reveals localization of ω-N_3_-Sphingosin in *N. meningitidis*. (**a**,**b**) Representative CLEM images of *N. meningitidis* treated with 0.1 × MBC (**a**) or 1 × MBC (**b**) of ω-N_3_-Sphingosine. Upper rows show representative single images of the ω-N_3_-sphingosine [ω-N_3_-Sph (green)] and DNA signal (magenta) taken with SIM (**i**,**iii**) and *N. meningitidis* image taken with SEM (**ii**). Final images after unbiased correlation are shown in (**iv**). Scale bars 1 µm.

Next, bacteria were treated with a low concentration of ω-N_3_-sphingosine (0.1 × the MBC) and subsequently labeled with AFDye 488 DBCO AF and imaged by CLEM. Treatment of *N. meningitidis* with 0.1 × the MBC of ω-N_3_-sphingosine showed a similar morphological pattern compared to bacteria treated with ω-N_3_-C_6_-ceramide. Again, only a minor proportion of bacteria was affected and some showed elongations of the outer membrane, whereas most of the bacteria remained unaltered in their morphology and showed continuous intracellular content (Fig. 4A-ii,iii).

CLEM imaging showed that the functionalized sphingolipid integrated into the membrane extensions and could barely be found in the cytosol of the bacteria (Fig. 4a-i,iv). When *N. meningitidis* were treated with 1 × the MBC of ω-N_3_-sphingosine and subsequently labeled with the dye, bacteria displayed a similar morphology compared to bacteria treated with the functionalized ω-N_3_-C_6_-ceramide without dye labeling and analyzed by SEM (Fig. 4b). Bacteria appeared in various stages of lysis with compromise of the cell wall, outer and inner membrane and demonstrated an altered shape compared to control bacteria (Fig. 4b-ii,iii). By SEM we observed a strong accumulation in the electron dense regions in the cytosol of the bacteria. By CLEM imaging we could clarify that the electron dense region correlated to staining with and accumulation of the dye labeled ω-N_3_-sphingosine (Fig. 4b-i,iv).

### Functionalized sphingolipids primarily affect the outer membrane of *N. meningitidis* as confirmed by mass spectrometry

**Figure 5:**
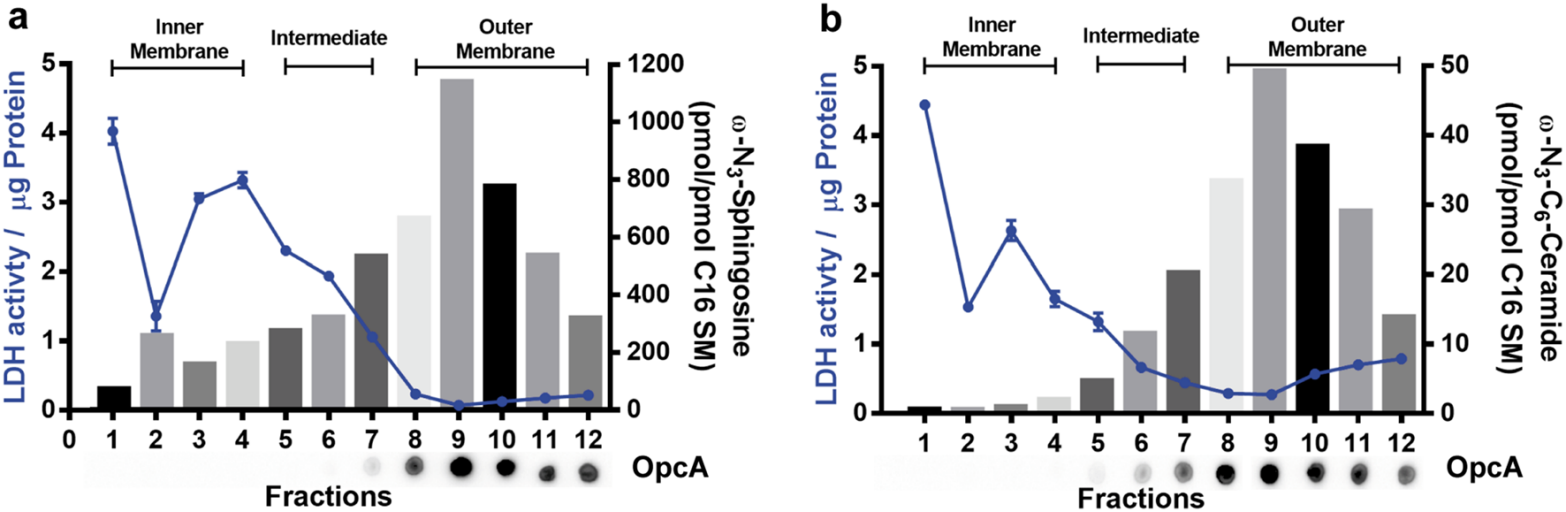
Quantification of azido-functionalized sphingolipid levels in membrane fractions of *N. meningitidis* by mass spectrometry. Characterization of membrane fractions (1–12) of *N. meningitidis* after treatment of bacteria with either ω-N_3_-sphingosine (**a**) or ω-N_3_-C_6_-ceramide (**b**). Each fraction was tested for LDH activity per µg protein (blue line), as inner membrane marker and OpcA abundance by dot blot (below X-axis) as outer membrane marker. Bars represent the amount of azido-functionalized sphingosine or ceramide, as determined by HPLC–MS/MS, normalized to the amount of C16 sphingomyelin (SM) found in the samples. LDH data show the mean ± SD of a representative experiment performed in duplicate.

Due to the lower resolution of the fluorescence imaging, compared to the electron microscopy, we aimed to confirm the indicated localization in the outer membrane, after low concentration treatment, by HPLC–MS/MS. For that, we separated the inner and outer membrane of the bacteria after incubation with 0.1 × the MBC of either ω-N_3_-sphingosine or ω-N_3_-C_6_-ceramide and quantified the amount of functionalized lipids in the different membrane fractions (1–12). Both treatments show a similar picture in which the lactate dehydrogenase (LDH) activity, a marker for the inner membrane, decreases with the increasing fractions number (from 1 to 12), whereas the amount of the outer membrane protein OpcA increased (Fig. 5a,b). The amount of ω-N_3_-sphingosine (Fig. 5a) or ω-N_3_-C_6_-ceramide (Fig. 5b), measured in the same fractions that have been used for the fraction characterization, show a negative correlation with the LDH activity and a positive correlation with the OpcA amount. Classification of the fractions into inner membrane, intermediate and outer membrane was done with the ethanol (EtOH) treated control group, based on the LDH activity, and transferred to the treated samples (Fig. S4).

For ω-N_3_-sphingosine and ω-N_3_-C_6_-ceramide, fraction number 9 seemed to be the cleanest outer membrane fraction with the lowest LDH activity and the strongest OpcA signal. In this specific fraction, the amount of functionalized lipids was strongly increased, especially compared to the inner membrane fractions indicating the primary effect on the outer membrane. Of note, functionalized sphingolipids were quantified in relation to the canonical sphingolipid C16 sphingomyelin (C16 SM), a typical membrane lipid whose occurrence in membrane fractions is most likely due to the medium used for cultivation of *N. meningitidis* strain MC58 (PPM+). HPLC–MS/MS experiments reveal that the medium contained 1.79 ± 0.01 pmol/ml C16 SM. Therefore, bacteria of the membrane separation experiment were exposed to 179 pmol C16 SM via the cultivation medium. It therefore seems plausible that the bacteria have absorbed the C16 SM detected in the membrane fractions (0.38–4.80 pmol/ml) from the medium and integrated it into their membranes. For an endogenous synthesis of sphingomyelins in *N. meningitidis* there is no evidence in the literature.

## Discussion

In this study we observed that beside ω-N_3_-C_6_-ceramide^16^ an azido-modified sphingosine, ω-N_3_-sphingosine^22^, exhibited significant bactericidal activity. We show that an observable effect on bacteria of both modified sphingolipid analogs is distortion of the cell membrane. The introduced srAT technology in combination with click chemistry-based labeling reaction (Click-AT-CLEM) clearly revealed that the azido-modified sphingolipids localize to the outer membrane resulting in strong elongation of the outer membrane. Pre-embedding labeling as used in this study added another level of versatility to the srAT workflow. It enabled super-resolved localization of incorporated fluorescent tags in the full ultrastructural background without on-section labelling that is normally necessary in AT workflows^25,26^. This adds a level of simplification to the protocol as on section labelling steps are typically laborious and can be error prone^28–30^. To verify our findings, we separated the outer and inner membrane of bacteria after treatment with ω-N_3_-C_6_-ceramide or ω-N_3_-sphingosine for qualitative observations by quantitative mass spectrometric measurements. Indeed, HPLC–MS/MS results confirmed the primarily effect of the azido-modified sphingolipids on the outer membrane of *N. meningitidis*.

Human lipids with antimicrobial properties include free fatty acids, monoglycerides and sphingolipids, and their activity against Gram-positive and Gram-negative bacteria have been demonstrated in various studies^3,4,8–13^. Sphingolipids, including ceramides, form a diverse group of structurally related lipids and are composed of a backbone of sphingoid bases coupled to a fatty acid side chain. A broad range of different head motives and complex glycosylation pattern results in further variability^1^. Sphingoid bases and certain fatty acids are present in the oral mucosa and saliva. They are produced by either the oral epithelium or sebaceous glands. Due to their antimicrobial activity, recent work suggested that these lipids are also likely involved in innate immune defense against mucosal microorganisms.

The nasopharynx is the site of colonization by meningococci and the primary site of invasion prior to the development of systemic infection, such as sepsis and meningitis. Meningococci adhere to the nasopharyngeal mucosa via interactions between the human epithelial cells and a variety of adhesins and invasins ^31–36^. It is likely that a number of factors that contribute to the integrity of the mucosal barrier and prevent both colonization and invasion and antimicrobial lipids may probably play a role in preventing colonization and invasion of these bacteria. We have recently analyzed the antimicrobial properties of sphingolipids against *N. meningitidis*^13^, including sphingosine, short-chain C_6_ and long-chain C_16_-ceramides as well as azido-functionalized ceramide analogs. We found that treatment with sphingosine, short-chain C_6_ ceramide and ω-N_3_-C_6_-ceramide lead to efficient killing of *N. meningitidis*^13^*.* Of note*,* short-chain C_6_ ceramide and ω-N_3_-C_6_-ceramide were inactive against *E. coli* and *S. aureus*^13^. Here, we now extended the findings of our previous study and included an azido-functionalized sphingosine to determine its antimicrobial activity against *N. meningitidis.* ω-N_3_-Sphingosine displayed significant antimicrobial activity against *N. meningitidis,* and MIC/MBC values were comparable to MIC/MBC values displayed for the unmodified sphingosine.

However, while the inhibitory activity of those antimicrobial sphingolipids has been investigated for a longer time, using biological approaches by us and other researchers^3,4,13^, the exact mechanism of the antibacterial activity on the bacterial cell was only recently investigated for the natural sphingosine^14^. The authors showed a massive increase in membrane permeability in *Pseudomonas aeruginosa* and *S. aureus* after treatment with sphingosine and linked this to the interaction between the protonated form of the sphingosine NH_2_ group and the highly negatively charged bacterial membrane lipid cardiolipin. However, because ceramides including ω-N_3_-C_6_-ceramide lack this specific NH_2_ group, due to the fatty acid side chain, the mode of action of ω-N_3_-C_6_-ceramide remained elusive.

Gram-positive bacteria are characterized by having a cytoplasmatic membrane and a thick peptidoglycan cell wall, which confers the characteristic cell shape and provides the cell with mechanical protection^37^. Gram-negative bacteria such as the β-proteobacterium *N. meningitidis* are characterized by the presence of two distinct membranes, called inner and outer membrane and a thin peptidoglycan cell wall between them^37^. The bacterial cell membranes are mainly formed by polar lipid bilayers (e.g. phospholipids) and it is likely that sphingolipids may insert into the outer membrane or the cytoplasmatic membrane of Gram-negative bacteria. Insertion into bacterial membranes may directly change the physical properties of the bacterial membrane and render the membrane non-functional. Alternatively, sphingolipids may penetrate and accumulate in the cytoplasm and may interfere with the cell metabolism. To address the question of the mode of action, electron microscopy (EM) has been widely used to visualize the antibacterial activity of antimicrobial lipids by determining morphological changes after treatment of bacteria with the respective antimicrobial lipid. Most studies utilize transmission electron microscopy (TEM) for imaging the effects of lipids^38–43^, since this technique allows the characterization of surface morphology along with the density of inner cytoplasmatic constituents, presence of fibers or cell vacuolization. Here we first visualized the effect of two azido-modified sphingolipids on *N. meningitidis* by scanning electron microscopy (SEM) and TEM. Treatment of *N. meningitidis* with 0.1 × MBC of both compounds showed strong elongation of the outer membrane, however the cell wall and intracellular content were intact. When *N. meningitidis* were treated with 1 × MBC of both compounds, bacteria showed different stages of disintegration and lysis. The cytoplasm was not uniform with flocculation and aggregation of intracellular contents. Bacteria showed electron dense intracellular inclusion bodies and additional vesicles that filled the intracellular content. Interestingly, our results are in line with recent published data observed for *E. coli* and *S. aureus* after treatment with different sphingoid bases^12^. In a study by Fischer and colleagues the authors examined the effects of different sphingoid bases, including sphingosine, dihydrosphingosine and phytosphingosine, on *E. coli* and *S. aureus* in detail by TEM and SEM^3,12^. Sphingosine treated cells of both *E. coli* and *S. aureus* contained electron dense intracellular bodies similar to treatment with the azido-modified sphingosine as reported in our study. Moreover, treatment of *E. coli* with unmodified sphingosine resulted in surface bleb formation, while the cell wall appeared to be intact^12^. Our results observed for treatment of *S. aureus* with the azido-modified sphingosine is also similar to those obtained when cells are treated with the unmodified sphingosine: bacteria are also in various stages of disintegration and lysis and cellular debris are clearly visible near damaged cells^12^.

It is interesting to note that azido-modified ceramides had no growth inhibitory effect on *E. coli* or *S. aureus*^13^. In case of *S. aureus*, this finding might be explained due to the fact that azido-modified ceramides initially integrate into the outer membrane—which is absent in Gram-positive bacteria—to exhibit toxic effects or that azido-modified ceramides cannot pass the thick peptidoglycan cell wall to interact with the cytoplasmatic layer. In case of *E. coli*, we tested the hypothesis that due to the presence of the complex glycolipid, the lipopolysaccharide (LPS), in the outer leaflet of the lipid bilayer, azido-modified ceramides may be hindered to intercalate into the outer membrane. In contrast to *E. coli*, *N. meningitidis* expresses a lipooligosaccharide (LOS), lacking the O-antigen of the classical LPS^44^. We therefore tested an *E. coli* K12 strain MG1655, lacking the O-antigen^45,46^ for its susceptibility to treatment with azido-modified ceramides and determined MIC and MBC values. For *E. coli* K12 strain MG1655, lacking the O-antigen, MIC/MBC values ˃ 64 µg/ml were determined and demonstrated that bacteria are still resistant (Table S1).

Antibody based CLEM approaches for precise localization of epitopes of interest in the ultrastructural context combine the advantages of light and electron microscopy. Said that, often the limit is the antibody itself due to multitude of possible technical obstacles, such as weakness of interaction in the EM-sample preparation steps, pure size of the antibody that hinders precise localization or, in the worst case, complete lack of a suitable antibody for a given epitope. The click-chemistry approach paves the way to circumvent several of these obstacles of antibody based CLEM approaches as successfully shown for click-labelling on cryosections^47^. Although a big breakthrough in CLEM, labelling of sections, be it cryo or resin-sections, is often laborious and technically challenging. To circumvent these limits of on-section labelling, we established a novel pre-embedding labelling protocol based on super-resolution array tomography (srAT)^17,18,25,26^ and click chemistry reaction^19^ for fluorescence and electron imaging of azido-modified sphingolipid analogs on *N. meningitidis.* The improved srAT-CLEM approach (Click-AT-CLEM), developed for this study, combines the strength of classical AT-CLEM protocols with the advantages of click chemistry^18,48^. The possibility of epitope localization within the EM resolution range is strongly improved by the unmatched specificity of click chemistry reactions. In the classical, antibody-based AT approach, post fixation and embedding staining leads to a reduction of accessible epitopes^27^. Due to the pre-fixation and embedding staining in the novel Click-srAT protocol, we achieved an accessibility of all surface epitopes thus leading to an improved signal with less hand on time.

Our CLEM studies confirmed the results of the morphological studies und clearly showed the incorporation of azido-modified sphingolipids into the outer membrane of *N. meningitidis*. This novel pre-embedding Click-srAT protocol has the advantage that it can be combined with post-embedding antibody staining as in classical AT and srAT. This adds another level of versatility to our novel approach. Notably, as the clicked dye is integrated within the sections and not only on the surface the strength of the signal can be increased by turning to thicker sections if the detection of the signal is limiting and thereby adding up signal throughout the thickness of the section. Furthermore, also the fluorescent channels could be potentially switched or even combined in a multi-channel pre-embedding labelling approach, as we could show that also rhodamine stays fluorescent in the LR-White resin throughout the embedding steps^49^. An important point in qualitive experiments, like the established Click-AT-CLEM approach, is the proof of observation by quantitative experiments. For that purpose, we successfully separated the inner (fractions 1–4) and outer membrane (fractions 8–12) of *N. meningitidis* after treatment with the functionalized lipids, as shown by the comparison of LDH activity and OpcA abundance, and used these samples for mass spectrometric analyses (Fig. 5). The results clearly showed a pronounced increase in concentration with fraction 9 being the one with the highest observed amount of both functionalized sphingolipids. Remarkably, this fraction appeared to be the cleanest outer membrane fraction with a strong OpcA signal and the lowest LDH activity. These results confirm our observations during the Click-AT-CLEM observations and emphasize the primary effect of the outer membrane during low concentration treatment. This novel approach complements biological approaches, such as growth inhibitory assay and SEM or TEM, and enables to decipher the mechanism of the antibacterial activity of sphingolipids.

## Methods

### Bacterial strains

*Neisseria meningitidis* strain MC58 (a serogroup B strain of the sequence type (ST)-74 (ST-32 clonal complex [cc])) was used in this study. This strain was isolated in the UK (1983) and was kindly provided by Moxon^50^. *N. meningitidis* MC58 was grown overnight on Columbia blood agar plates (bioMérieux) at 37 °C in 5% CO_2_ and cultured on the next day in PPM+ (proteose-peptone medium (PPM) supplemented with 1 × Kellogg’s supplement, 0.01 M MgCl_2_, and 0.005 M NaHCO_3_ (PPM+)). *E. coli* (ATCC 25922, Serotype 06; Biotype 1), *E. coli* K12 (MG1655) and *S. aureus* (ATCC 29213, subspecies *aureus* Rosenbach) were cultivated overnight at 37 °C with shaking in lysogenic broth (LB) for *E. coli* or tryptic soy broth (TSB) for S*. aureus* liquid culture.

### Lipids

d-*erythro-*Sphingosine was purchased from Santa Cruz Biotechnology (Heidelberg, Germany). ω-N_3_-C_6_-ceramide (d18:1/6:0-ω-N_3_) (6-azido-*N*-((2*S*,3*R*,*E*)-1,3-dihydroxyoctadec-4-en-2-yl)hexanamide) was synthesized by amide coupling of sphingosine according to literature^16^. ω-N_3_-sphingosine ((2*S*,3*R*,*E*)-2-amino-18-azidooctadec-4-ene-1,3-diol) was chemically synthesized as previously described^22^. All lipids were dissolved in pure ethanol and stored at − 20 °C.

### Antimicrobial assay

Minimal inhibitory and bactericidal concentration (MIC and MBC) values were estimated using broth microdilution assay. The assays were performed as described elsewhere with minor changes^13^. Briefly, lipids were geometrically diluted in either PPM+ for *N. meningitidis* or Müller-Hinton broth (Difco Laboratories) for *E. coli* and *S. aureus*. Concentrations from 128 to 1 µg/ml were diluted 1:1 with 1 × 10^8^ bacteria/ml in the corresponding medium resulting in test concentration ranging from 64 to 0.5 µg/ml. At concentration higher than 64 µg/ml the lipids had an optical density that interfered with the determination of the MIC. Plates were incubated for 16 h at 37 °C and 5% CO_2_ (for *N. meningitidis)* and 37 °C (for *S. aureus* and *E. coli*). Afterwards, the optical density of bacterial growth was read at 540 nm in a spectrophotometer (Infinite F200 Pro Reader, Tecan Group, Maennedorf, Switzerland) or MIC was directly read from the plate. The MIC was defined as the lowest concentration at which no visible growth occurs, and the MBC was defined as the concentration of lipid that prevented growth. Quality control was monitored with *E. coli* ATCC 25922 and *S. aureus* isolates ATCC 29213.

### Transmission electron microscopy (TEM)

1 × 10^9^ bacteria from a pre-culture were inoculated into 10 ml of fresh PPM+ containing either 0.1 or 1 × the MBC concentration of the compound tested. After 3 h of incubation at 37 °C in a shaking incubator, the bacteria were harvested for 15 min at 4000*g*. The pellet was then washed once with phosphate buffered saline (PBS), resuspended in 100 µl of fixation solution (2.5% glutaraldehyde in 50 mM cacodylate buffer), centrifuged and incubated for 1 h at room temperature (RT). Afterwards, the bacteria pellet was washed 5 times (15 min per wash) with cacodylate buffer (50 mM KCl, 2.5 mM MgCl_2_, 50 mM cacodylate, pH 7.2) and post fixed with osmium tetroxide (2% OsO_4_ in cacodylate buffer) for 2 h. After 5 washing steps with water, additional *en bloc* contrast was achieved by incubating the samples in 0.5% uranyl acetate (in dH_2_O) overnight. Following 5 washing steps, the samples were dehydrated in a gradient of increasing ethanol concentrations (50, 70, 90% in H_2_O and 3 × 100%; 30 min each) and subsequent incubation in 100% propylene oxide (two times, 30 min each). The samples were infiltrated with 50% Epon resin in propylene oxide overnight. Next, they were transferred to 100% Epon which was changed two times after 2 and 4 h and cured at 60 °C for 48 h. Ultrathin sections (70 nm) were cut using the Leica EM UC7 ultramicrotome, contrasted with uranyl acetate and lead citrate following a standard procedure and images were taken with the JEOL JEM-2100 transmission electron microscope (JEOL Germany, Freising, Germany) at 200 kV. Images were recorded with a TemCam-F416 (TVIPS, Gauting, Germany) digital camera.

### Scanning electron microscopy (SEM)

Bacteria were treated as described above. After incubation with the compounds, bacteria were harvested, resuspended in 1 ml PPM+ and applied to poly-l-lysine coated round cover glasses for 2 h. The supernatant was then removed and the cover glasses were washed once with 1 ml PBS. Afterwards, fixation was performed with 6.25% glutaraldehyde in Sörensen buffer (pH 7.4: 82 ml of 133 mM Na_2_HPO_4_ in water, filled up to 100 ml with 133 mM KH_2_PO_4_) overnight at 4 °C. After 5 washing steps with Sörensen buffer, the samples were dehydrated with increasing acetone concentrations (30, 50, 75, 90% in H_2_O and 100%, 30 min each). Then, critical point drying was performed using the Critical Point Dryer CPD030 (Baltec). The dried samples were coated with a thin gold–palladium (80:20) coating using the Sputter Coater SCD005 (Baltec). Images were taken with the JSM-7500F scanning electron microscope (JEOL Germany, Freising, Germany).

### Correlative light and electron microscopy (CLEM) and click chemistry

The CLEM workflow was adapted from the super-resolution array tomography protocol (srAT) described previously^17,18^ with certain changes. Bacteria were grown and treated as described in the TEM methods section. Afterwards, the cell pellet was resuspended in 100 µl of 5 µM Dibenzocyclooctyne-amine Alexa Fluor 488 (DBCO-AF488; Jena Bioscience, Germany) and the click reactions between the functional azide group of the lipid and the functional DBCO group of the dye took place at 37 °C for 15 min. After washing once with PBS, the pellet was fixed in 4% formaldehyde (in PBS) for 1 h at RT. The pellet was washed 5 times in PBS and incubated in ammonium chloride (50 mM ammonium chloride in PBS) for 15 min. Following several washes in water, the samples were embedded in LR White resin according to the following Progressive Lowering of Temperature (PLT) protocol. The initial dehydration step of 30% ethanol in water happens on ice (2 × 15 min), then the following steps (50%, 70% (with 0.2% uranyl acetate), 90% (with 0.2% uranyl acetate) and 100% ethanol; 2 times 30 min each) at -20 °C. A mixture of 100% ethanol and LR White (1:1) was incubated overnight and resin infiltration was performed within 24 h, with washes after 1 h, 4 h, 20 h and 24 h at 4 °C. The resin was polymerized at 42 °C for 3 days. Ultrathin sections (100 nm) were transferred onto a poly-l-lysine coated microscopy slide and stained with the DNA dye methyl green (1:10,000 in dH_2_O from a 2% stock in water) for 10 min, rinsed off and mounted in Mowiol and high precision cover glasses^51^. Fluorescence images were acquired using the Elyra S.1 SIM (Zeiss, Germany). The samples were then processed for imaging at the SEM by removing the cover glass, washing off the mounting medium and contrasting with heavy metals as follows: the sections are first incubated in 2.5% uranyl acetate in ethanol for 15 min and in 50% lead citrate in H_2_O (according to Reynolds’ ^52^) for 10 min. Carbon coating was performed with the Compact Coating Unit CCU-010 (Safematic, Bad Ragaz, Switzerland) resulting in a ca. 3 nm coating. Images were taken with the JSM-7500F scanning electron microscope (JEOL USA, Inc., Peabody, MA USA).

Registration of the fluorescence and electron microscopy images was accomplished with Inkscape 0.91 (Inkscape Community) using the heterochromatin pattern as an intrinsic landmark in both channels (for a detailed description see^17,18^. For an unbiased correlation the channel of interest (lipid signal) was hidden and only the DNA signal was used. After the correlation the channel of interest was revealed, and all channels could be merged.

### Neisserial membrane separation

Membrane separation of the meningococcal outer and inner membrane (OM/IM) was performed with minor changes, as previously described^53^. Briefly, either ω-N_3_-C_6_-ceramide, ω-N_3_-sphingosine or a corresponding amount of EtOH were added to a final concentration of 0.1 × the MBC to 100 ml cultures of *N. meningitidis* strain MC58 with an OD600 of 0.1. After 3 h of incubation at 37 °C in an orbital shaker, bacteria were harvested and washed three times with PBS and in the end resuspended in 50 mM Tris–HCl (pH 8). Afterwards, lysozyme (100 µg/ml) and EDTA (pH 8, 5 mM) were added and the bacteria were incubated for 1 h at RT while shacking. This step was followed by one freeze thaw cycle (− 80 °C to 37 °C) and sonication (10 s pulses with 5 s breaks, at 100% magnitude for a total of 5 min). Unbroken bacteria were removed by centrifugation at 1200*g* and 4 °C for 10 min. Then, the supernatant was loaded on top of a sucrose gradient, consisting of a 3 ml 15% top layer over a 2 ml 55% cushion (both with 5 mM EDTA pH 8), and centrifuged at 280,000*g* and 4 °C for 2 h. The crude membrane fraction was then collected, the sucrose concentration estimated with a refractometer and lowered to 30% by dilution with dH_2_O (with 5 mM EDTA, pH 8). Afterwards, the sample was loaded onto a sucrose gradient consisting of 1.3 ml 50% cushion and 45, 40, 35% layer on top (2.4 ml each). The gradient was then centrifuged for 41 h at 268,000*g* and 4 °C and afterwards 800 µl samples were collected from top to bottom.

Then, the purity of the isolated OM and IM fraction was analyzed. Lactate dehydrogenase (LDH) was chosen as inner membrane marker^54^ and the presence of the meningococcal OpcA protein as outer membrane marker. LDH was determined with a commercial LDH activity assay and used according to the manufacturer’s instructions (Sigma Aldrich). Activity was calculated as LDH activity/µg protein (determined by Bradford assay). To visualize the relative amount of OpcA in the samples, the protein amounts were adjusted and utilized in a dot blot assay with a monoclonal mouse anti-OpcA antibody (clone B306, kindly provided by M. Achtman). 4 µl of each sample was spotted onto a nitrocellulose membrane. After drying, nonspecific binding sites were blocked with 5% skim milk in PBS supplemented with 0.05% Tween 20 (PBS-T) for 1 h at RT on an orbital shaker. Afterwards, primary antibody incubation was carried out with a 1:10,000 dilution of the anti-OpcA antibody for 30 min followed by three washing steps with PBS-T (5 min each). For secondary antibody incubation, an anti-mouse IgG antibody, conjugated to horseradish peroxidase (HRP), was used with a 1:1000 dilution in 5% skim milk in PBS-T. After 30 min incubation at RT, the membrane was washed again three times with PBS-T and once with PBS for 5 min each. Finally, ECL substrate (BioRad) was added for 1 min and the protein was visualized using the ChemiDoc MP Gel and Blot Imaging System (BioRad).

### Quantification of azido-functionalized sphingolipid derivatives by HPLC–MS/MS

Samples of neisserial membrane fractionation (600 µl) were filled up with water to 1 ml followed by addition of 110 µl 10 × Baker buffer (300 mM citric acid, 400 mM disodium hydrogen phosphate, pH 3.0). For lipid extraction, 2 ml 1-butanol and 1 ml water-saturated 1-butanol were added. The extraction solvent contained sphingosine-d_7_ (Sph-d_7_), sphingosine 1-phosphate-d_7_ (S1P-d_7_), C17 ceramide (C17 Cer) and C16 sphingomyelin-d_31_ (C16 SM-d_31_) (all Avanti Polar Lipids, Alabaster, USA) as internal standards. Extraction was facilitated by intensive vortexing (1500 rpm) for 10 min at RT. Afterwards, samples were centrifuged for 5 min at 2200*g* (4 °C). The upper organic phase was dried under reduced pressure using a Savant SpeedVac concentrator (Thermo Fisher Scientific, Dreieich, Germany). Dried residues were reconstituted in 200 μl acetonitrile/methanol/water (47.5:47.5:5 (v:v:v), 0.1% formic acid) and subjected to HPLC–MS/MS sphingolipid quantification. Chromatographic separation was achieved on a 1260 Infinity HPLC (Agilent Technologies, Waldbronn, Germany) equipped with a Poroshell 120 EC-C8 column (3.0 × 150 mm, 2.7 µm; Agilent Technologies) guarded by a pre-column of identical material. MS/MS analysis was carried out using a 6490 triple-quadrupole mass spectrometer (Agilent Technologies) operating in the positive electrospray ionization mode (ESI+). Long-chain bases (dihydro-Sph, Sph, S1P), Cer and SM species (C16, C18, C20, C22, C24 and C24:1) were analyzed by selected reaction monitoring (SRM) as described recently^55^. Additionally, the following mass transitions were recorded for quantification of functionalized sphingolipids (collision energies (CE) in parentheses): *m/z* 341.3 → 323.3 for ω-N_3_-Sph (8 eV) and *m/z* 439.4 → 421.4 (15 eV), *m/z* 439.4 → 282.3 (25 eV), *m/z* 421.4 → 264.3 (25 eV, quantifier) for ω-N_3_-C_6_ Cer (d18:1/6:0-ω-N_3_). Sph-d_7_ and C17 Cer served as internal standards for quantification of ω-N_3_-Sph and ω-N_3_-C_6_ Cer, respectively. Moreover, quantities of azido-functionalized sphingolipid derivatives were normalized to the C16 SM (SM d18:1/16:0) content of each membrane fraction analyzed. Data processing was performed with MassHunter Software (Agilent Technologies).

### Statistical analysis

Statistical analysis was performed with GraphPad Prism 6 (GraphPad Software Inc., La Jolla, CA, USA) by analysis of variance (ANOVA) test followed by a post hoc test. Asterisks indicate significance values: *P < 0.05; **P < 0.01; ***P < 0.001; ****P < 0.0001.

## Supplementary Information

Supplementary Information

## Data Availability

All data generated or analyzed during this study are available from the author on reasonable request.
