# Supplementary material for: Click-correlative light and electron microscopy (click-AT-CLEM) for imaging and tracking azido-functionalized sphingolipids in bacteria
Source: Sci Rep. 2021 Feb 22;11:4300. doi: 10.1038/s41598-021-83813-w (PMC7900124; doi:10.1038/s41598-021-83813-w)
Supplement: Supplementary file 1 — Supplementary Information [file 41598_2021_83813_MOESM1_ESM.docx]

**Click-correlative light electron microscopy (Click-AT-CLEM) for imaging and tracking azido-functionalized sphingolipids in bacteria**

Simon Peters^1^, Lena Kaiser^1^, Julian Fink^2^, Fabian Schumacher^3,6,7^, Veronika Perschin^4^, Jan Schlegel^5^, Markus Sauer^5^, Christian Stigloher^4^, Burkhard Kleuser^3,6^, Jürgen Seibel^2^, and Alexandra Schubert-Unkmeir*^1^

^1^Institute for Hygiene and Microbiology, Julius-Maximilian University Wuerzburg, Wuerzburg, Germany

^2^Institute for Organic Chemistry, Julius-Maximilian University Wuerzburg, Wuerzburg, Germany

^3^Institute of Pharmacy, Freie Universität Berlin, Berlin, Germany

^4^Imaging Core Facility, Biocenter, Julius-Maximilian University Wuerzburg, Wuerzburg, Germany

^5^Department of Biotechnology and Biophysics, Biocenter, Julius-Maximilian University Wuerzburg, Wuerzburg, Germany

^6^University of Potsdam, Department of Toxicology, Nuthetal, Germany

^7^Institute of Molecular Biology, University of Duisburg-Essen, Essen, Germany

Keywords: *Neisseria meningitidis*, sphingolipids, antimicrobial, click chemistry, correlative light and electron microscopy (CLEM)

* Correspondence: Prof. Dr. Alexandra Schubert-Unkmeir

[aunkmeir@uni-wuerzburg.de](mailto:aunkmeir@uni-wuerzburg.de)

**Figure S1**


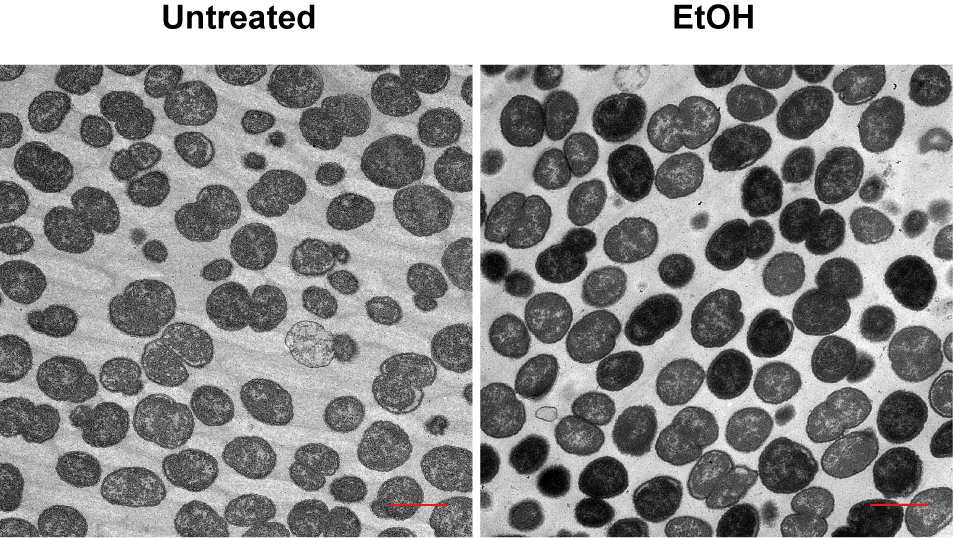


**Fig. S1: Ethanol treatment did not induce morphological changes in *N. meningitidis.***

Representative TEM images of *N. meningitidis* either left untreated (left) or treated with EtOH (right). Bacteria were treated with the highest amount of EtOH used as solvent in this study. Scale bars:1 µm.

**Figure S2**


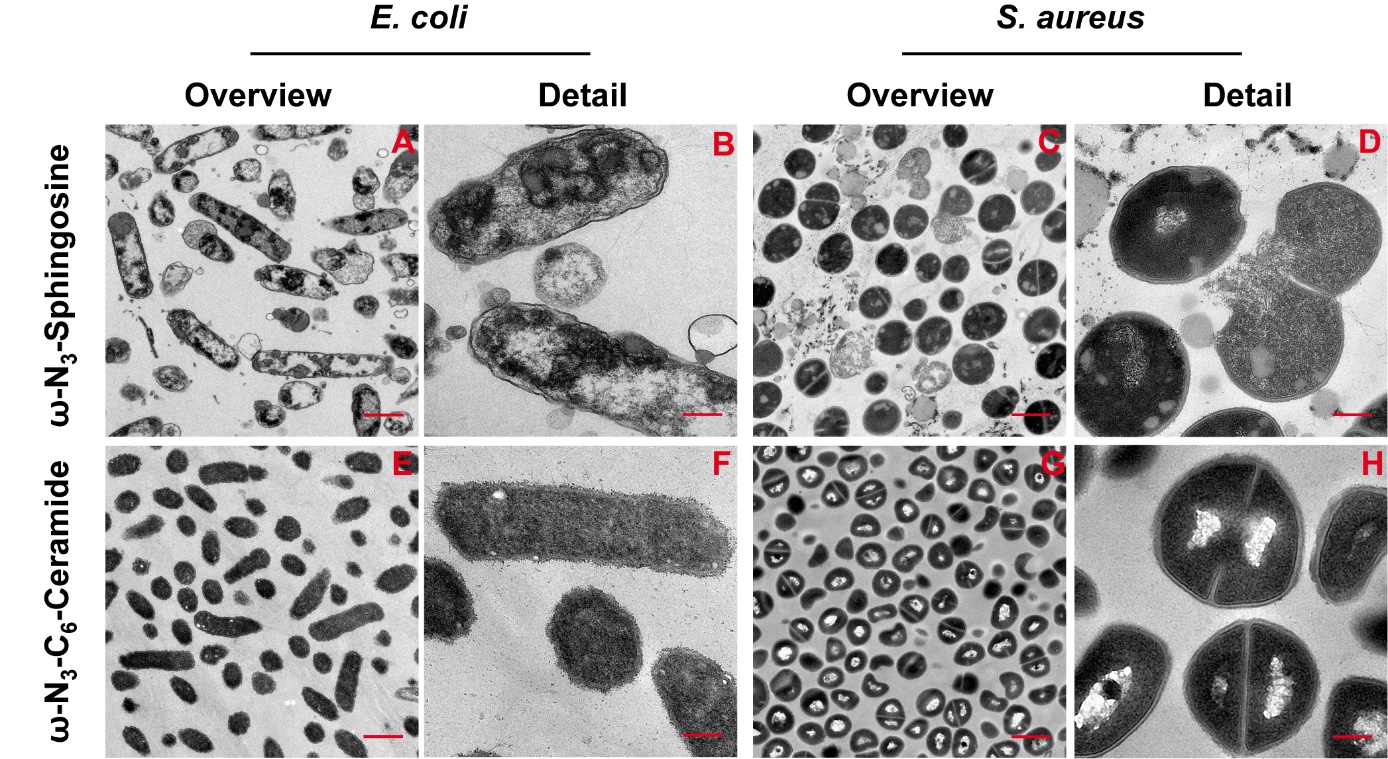


**Fig. S2: ω-N_3_-Sphingosine, but not ω-N_3_-C_6_-Ceramide has an effect on *E. coli* or *S. aureus* morphology.**

TEM studies of *E. coli* (left panel) and *S. aureus* (right panel) treated with 1 X MBC of ω-N_3_-sphingosine (A-D) or ω-N_3_-C_6_-ceramide (E-H). 1 X MBC corresponds to a value of 1 X MBC determined for *N. meningitidis* (= 4µg/ml)). Scale bars in overview images: 1 µm; Scale bars in detail images: 0.25 µm.

**Figure S3**


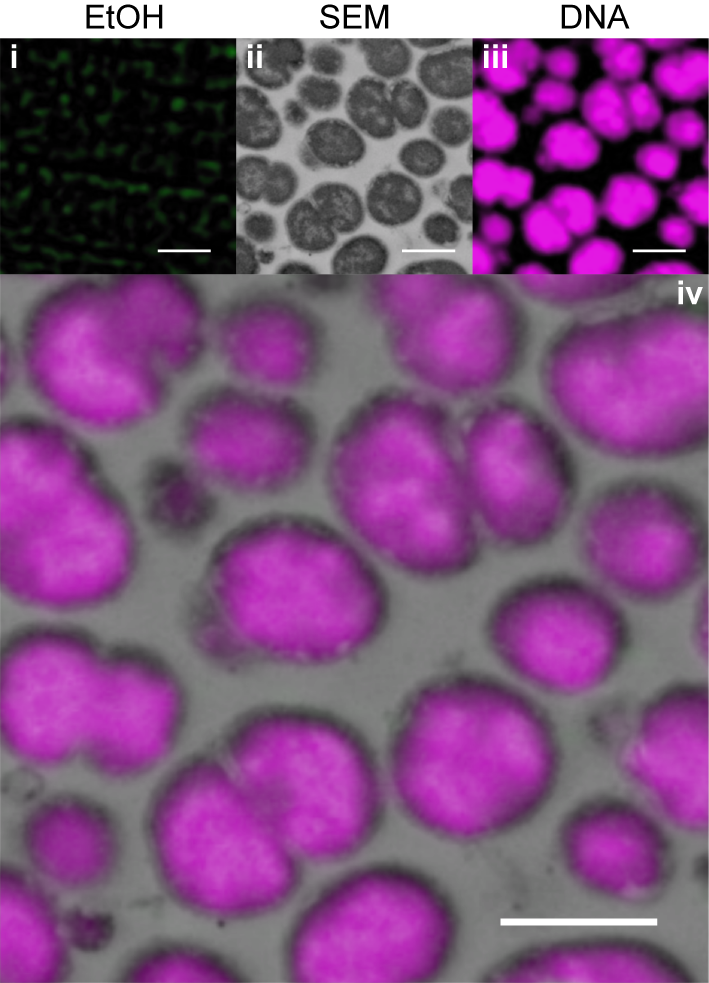


**Fig. S3: No unspecific binding of DBCO-AF-488 to *N. meningitidis.***

Representative CLEM image of *N. meningitidis* treated with EtOH (solvent control) and processed for CLEM, including labeling with AFDye™ 488 DBCO AF. Upper row shows representative single images of the AFDye™ 488 DBCO AF (green) and DNA signal (magenta) taken with SIM (i and iii) and *N. meningitidis* image taken with SEM (ii). Final image after unbiased correlation is shown in iv. Scale bars: 1 µm.

**Figure S4**


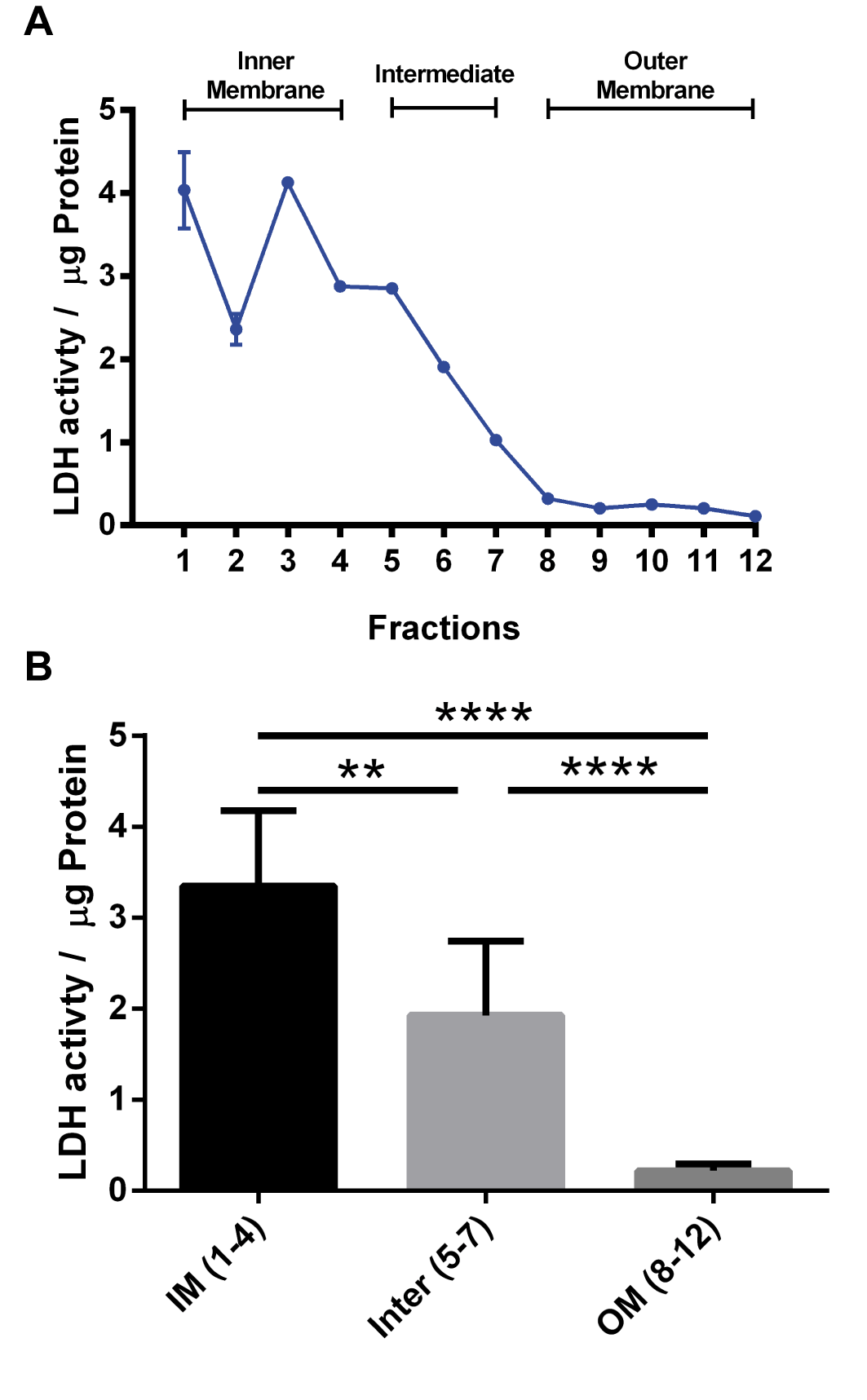


**Fig. S4: Analysis of fractions obtained after sucrose gradient centrifugation of *N. meningitidis* membranes based on LDH activity.**

Characterization of membrane fractions (1 – 12) of *N. meningitidis* after treatment with EtOH. (A) Each fraction was tested for LDH activity per µg protein (blue line) and separated into 3 groups (Inner Membrane, Intermediate, Outer membrane). The graph shows the mean ± SD of a representative experiment performed in duplicate. (B) Statistical comparison of the LDH activity between the pooled groups (Inner Membrane (IM) fractions 1 to 4, Intermediate (Inter) fractions 5 to 7 and Outer Membrane (OM) fractions 8 to 12). The graph shows the mean ± SD. One-way ANOVA with Dunnett’s post hoc test was used to determine significance. **, P < 0.01; ****, P < 0.0001.

**Table S1**

Minimal inhibitory and bactericidal concentrations (MICs/MBCs) of unmodified sphingosine, ω-N_3_-sphingosine or ω-N_3_-C_6_-ceramide against *N. meningitidis (*MC58*), E. coli* (ATCC 25922), *E. coli* K12 (MG1655) and *S. aureus* (ATCC 29213)*.*

| **Strain** | **Compound** | **MIC**  **[µg / ml]** | **MBC**  **[µg / ml]** | **Reference** |
| --- | --- | --- | --- | --- |
| N. meningitidis (MC58) | Sphingosine | 4 | 4 | Becam et al. 2017^2^ |
|  | ω-N_3_-Sphingosine | 4 | 8 | This study |
|  | ω-N_3-_C_6_-Ceramide | 2 | 4 | This study |
|  |  |  |  |  |
| E. coli (ATCC 25922) | Sphingosine | 16 | 32 | Becam et al. 2017^2^ |
|  | ω-N_3_-Sphingosine | 16 | 16 | This study |
|  | ω-N_3-_C_6_-Ceramide | >32 | >32 | This study |
|  |  |  |  |  |
| E. coli K12 | ω-N_3-_C_6_-Ceramide | >64 | >64 | This study |
|  |  |  |  |  |
| S. aureus (ATCC 29213) | Sphingosine | 8 | 32 | Becam et al. 2017^2^ |
|  | ω-N_3_-Sphingosine | 8 | 16 | This study |
|  | ω-N_3-_C_6_-Ceramide | >32 | >32 | This study |

1. Markert, S. M. et al. 3D subcellular localization with superresolution array tomography on ultrathin sections of various species. *Methods Cell Biol* **140**, 21-47 (2017).

2. Becam, J. et al. Antibacterial activity of ceramide and ceramide analogs against pathogenic Neisseria. *Scientific Reports* **7**, 17627 (2017).
